# Supplementary material for: Diversity and Toxigenicity of Fungi that Cause Pineapple Fruitlet Core Rot
Source: Toxins (Basel). 2020 May 21;12(5):339. doi: 10.3390/toxins12050339 (PMC7291148; doi:10.3390/toxins12050339)
Supplement: Supplementary file 1 [file toxins-12-00339-s001.pdf]

Supplementary Materials: Diversity and Toxigenicity of Fungi that Cause Pineapple Fruitlet Core Rot

Bastien Barral, Marc Chillet, Anna Doizy, Maeva Grassi, Laetitia Ragot, Mathieu Léchaudel, Noel Durand, Lindy Joy Rose, Altus Viljoen and Sabine Schorr-Galindo

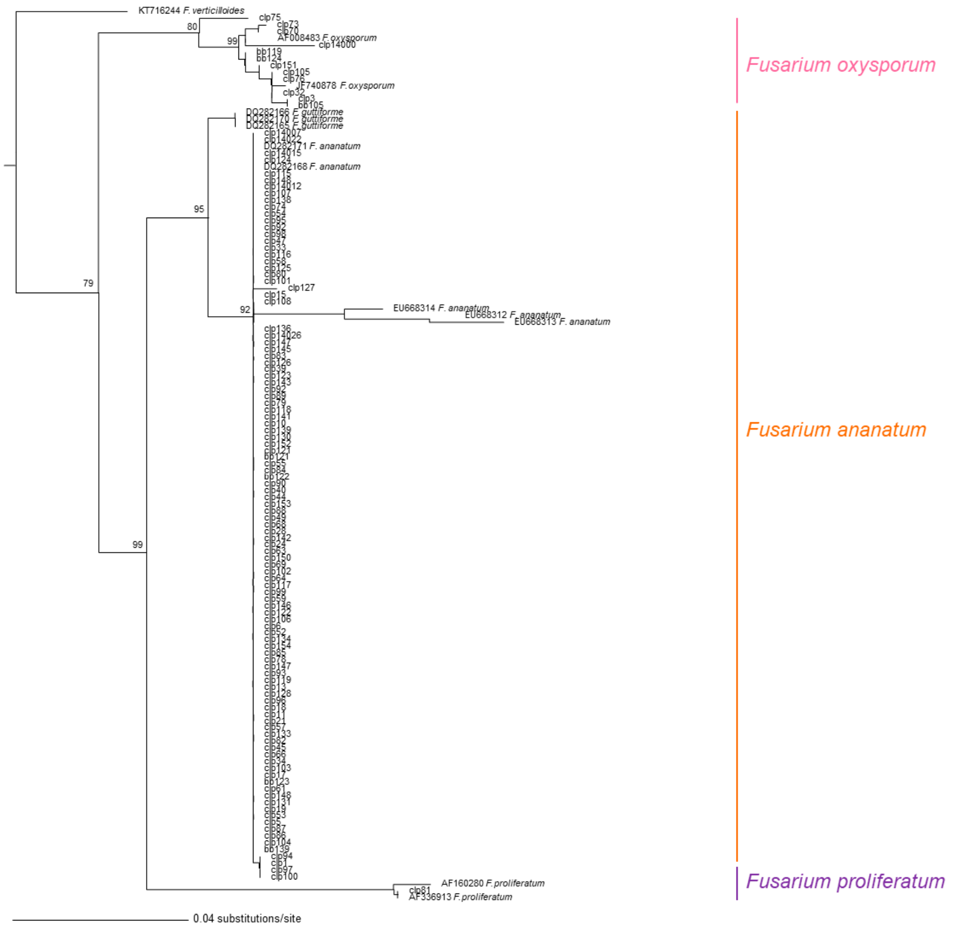

Figure S1. Maximum likelihood tree calculated using PhyML as based on translation elongation factor 1α (TEF-1α) gene sequences showing the relationships among *Fusarium ananatum* and related species. *Fusarium solani* was chosen as the outgroup. Bootstrap values (percentages of 100 bootstrap replicates) are indicated in brackets above the branches of the tree. Model selected: TN93 +G, alignment 709 bp.

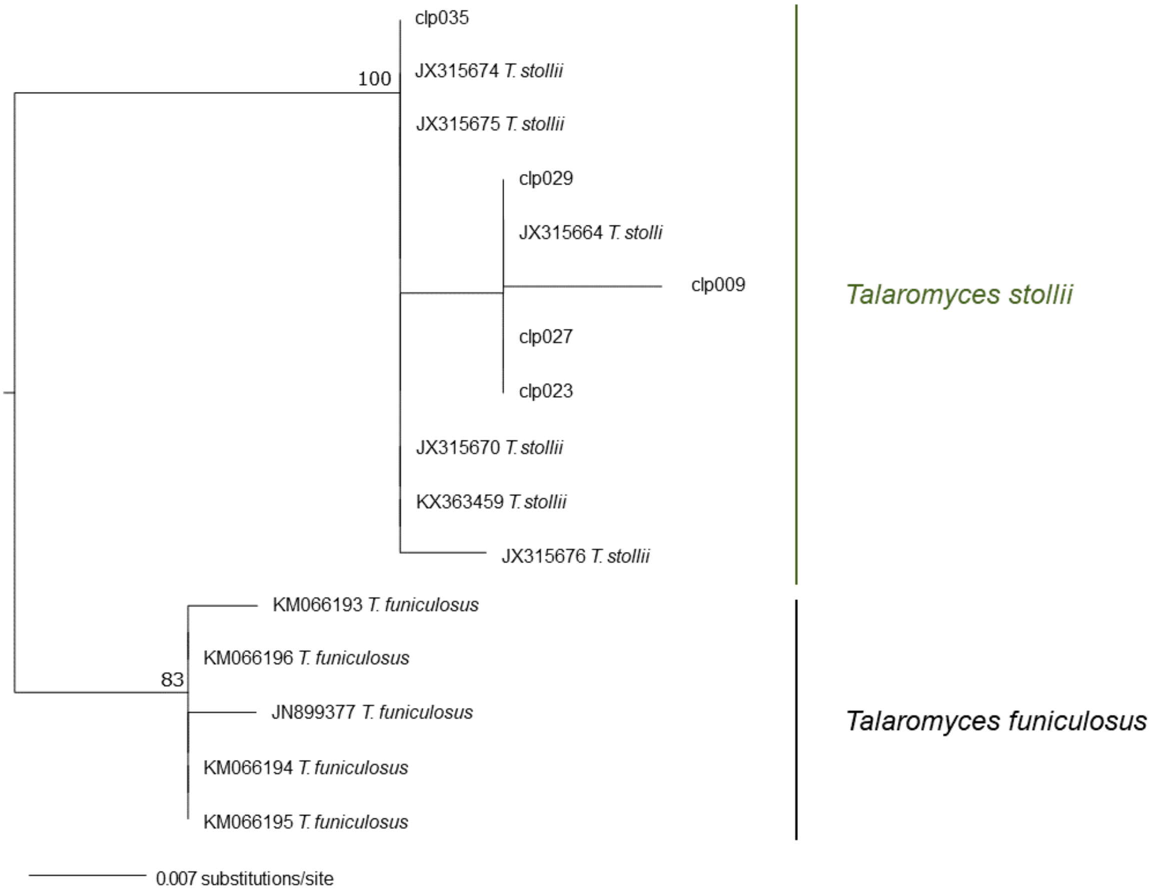

Figure S2. Maximum likelihood tree calculated using PhyML as based on internal transcribed spacer (ITS) sequences showing the relationships among *Talaromyces stollii* and related species. *Talaromyces dendriticus* was chosen as the outgroup. Bootstrap values (percentages of 100 bootstrap replicates) are indicated in brackets above the branches of the tree. Model selected: TN93, alignment 623 bp.
